# Global Adam17 Deficiency Preserves Renal Function and Modulates Integrated Pathogenic Responses in Experimental Diabetic Kidney Disease

**DOI:** 10.3390/ijms27146136

**Published:** 2026-07-09

**Authors:** Marta Riera, Claudia Martyn, Jordi Pujol-Brugués, Eva Márquez, Eva Rodríguez, Vanesa Palau, María José Soler, Javier Gimeno, Juan Sebastián Salazar Castañeda, Melissa Pilco, Jimena del Risco, Marta Crespo, Clara Barrios

**Affiliations:** 1 Hospital del Mar Research Institute, 08003 Barcelona, Spain; cmartyn@researchmar.net (C.M.); jpujol1@researchmar.net (J.P.-B.); eva.marquez.mosquera@hmar.cat (E.M.); erodriguezg@hmar.cat (E.R.); vpalau25@gmail.com (V.P.); mcrespo@hmar.cat (M.C.); 2 Nephrology Department, Hospital del Mar, 08003 Barcelona, Spain; juansebastian.salazar.castaneda@hmar.cat (J.S.S.C.); melissalorena.pilco.teran@hmar.cat (M.P.); jimena.delrisco.zevallos@hmar.cat (J.d.R.); 3 Nephrology Department, Hospital Universitari Vall D’Hebron, 08035 Barcelona, Spain; mariajose.soler@vallhebron.cat; 4 Department of Pathology, Hospital del Mar, 08003 Barcelona, Spain; jgimenobeltran@hmar.cat

**Keywords:** diabetic nephropathy, Adam17, inflammation, oxidative stress, fibrosis

## Abstract

Diabetic kidney disease (DKD) progression results from complex interactions between metabolic stress, inflammatory activation, maladaptive intracellular signalling, and fibrotic remodelling. While previous studies demonstrated renoprotective effects of cell-specific *Adam17* deletion, the impact of global *Adam17* deficiency on the integrated renal response to diabetes remains incompletely understood. Here, we investigated the effects of tamoxifen-induced global *Adam17* deletion in a streptozotocin-induced murine model of type 1 diabetes. Renal function, structural injury, inflammatory responses, stress-related signalling pathways, and fibrotic remodelling were comprehensively assessed in diabetic *Adam17* knockout and control mice. Despite persistent hyperglycemia and ongoing albuminuria, diabetic *Adam17* knockout mice exhibited preservation of glomerular filtration rate together with marked attenuation of diabetes-associated kidney injury. Global *Adam17* deletion reduced mesangial expansion and structural damage, limited macrophage infiltration and chemokine expression, and significantly attenuated fibrotic remodelling. At the molecular level, *Adam17* deficiency was associated with selective modulation of stress-related signalling pathways, including reduced activation of the PI3K/Akt axis and partial preservation of mitochondrial stress regulators, without evidence of generalized suppression of cellular stress responses. Notably, preservation of renal function occurred despite persistent albuminuria, supporting a partial dissociation between glomerular permeability alterations and progressive renal dysfunction. These findings demonstrate that global *Adam17* deletion confers robust protection against diabetes-associated kidney injury through coordinated attenuation of inflammatory, stress-related, and profibrotic pathways. Our results extend previous cell-specific observations and highlight the context-dependent role of Adam17 in DKD progression, supporting the concept that integrated Adam17-related signalling may represent a relevant therapeutic target in diabetic kidney disease.

## 1. Introduction

Diabetic kidney disease (DKD) remains a leading cause of chronic kidney disease and kidney failure worldwide, despite major advances in glycaemic control and the introduction of novel renoprotective therapies [1,2,3,4]. Although albuminuria has traditionally been considered a hallmark of diabetic nephropathy, progressive decline in glomerular filtration rate (GFR), structural kidney damage, and tubulointerstitial fibrosis are major determinants of long-term renal prognosis [1,5]. The increasingly recognised dissociation between albuminuria and renal function decline highlights the complexity of DKD progression and supports the need to identify molecular pathways that regulate kidney injury beyond glomerular permeability alone [1,5].

DKD progression results from the convergence of metabolic stress, inflammatory activation, maladaptive intracellular signalling, and fibrotic remodelling [4,6]. Sustained hyperglycemia promotes chemokine production, recruitment of inflammatory cells, oxidative and mitochondrial stress, and activation of profibrotic pathways within the kidney [3,4,6]. These processes do not occur in isolation but reflect dynamic interactions between resident renal cells, infiltrating inflammatory cells, and systemic metabolic cues [4,7,8,9]. Therefore, experimental approaches that capture integrated tissue responses may provide complementary information to cell-specific models.

A disintegrin and metalloproteinase 17 (Adam17), also known as tumor necrosis factor-α converting enzyme (TACE), is a membrane-bound sheddase that regulates the proteolytic release of cytokines, growth factors, and receptors, and adhesion molecules involved in inflammatory and growth-related signalling [10,11]. Through cleavage of substrates such as TNF-α, epidermal growth factor receptor (EGFR) ligands, and cytokine receptors, Adam17 has emerged as a central regulator of inflammatory and profibrotic pathways [12,13,14,15,16]. Hyperglycaemia, oxidative stress, and activation of the renin–angiotensin system have been shown to increase Adam17 expression and activity in renal cells. Enhanced Adam17-dependent shedding of TNF-α, EGFR ligands, and other inflammatory mediators has been implicated in the amplification of inflammatory and profibrotic responses characteristic of DKD, thereby contributing to renal injury progression. Consistent with these observations, increased Adam17 expression and activity have been reported in human diabetic nephropathy and experimental kidney disease, supporting its relevance in renal pathology [13,17,18]. Moreover, genetic variation and altered Adam17 expression or activity have been linked to inflammatory and metabolic phenotypes, including obesity and insulin resistance, supporting the biological relevance of Adam17 dysregulation in human disease [19,20,21].

Our group has previously shown that cell-specific *Adam17* deletion in renal proximal tubular epithelial cells or endothelial cells protects against kidney injury in experimental models of diabetes and metabolic disease [22,23,24]. These studies demonstrated that Adam17 contributes to renal damage through compartment-specific mechanisms involving inflammation, fibrosis and structural injury. However, DKD is a multifactorial and systemic disease [6,25,26,27], and the consequences of global *Adam17* deletion on the integrated renal response to diabetes remain incompletely defined. In particular, whether global *Adam17* deficiency can preserve renal function while modulating inflammatory recruitment, stress-related signalling, and fibrotic remodelling at the whole-kidney level has not been fully addressed.

In this study, we investigated the impact of tamoxifen-inducible global *Adam17* deletion in a streptozotocin (STZ)-induced model of type 1 diabetes. By integrating functional, histological, inflammatory, stress-related, and fibrotic analyses, we aimed to determine whether global *Adam17* deficiency protects against diabetes-associated kidney injury and to define the contribution of Adam17 to the coordinated pathological response driving diabetic nephropathy.

## 2. Results

### 2.1. Validation of Tamoxifen-Induced Global Adam17 Deletion

**Figure 1:**
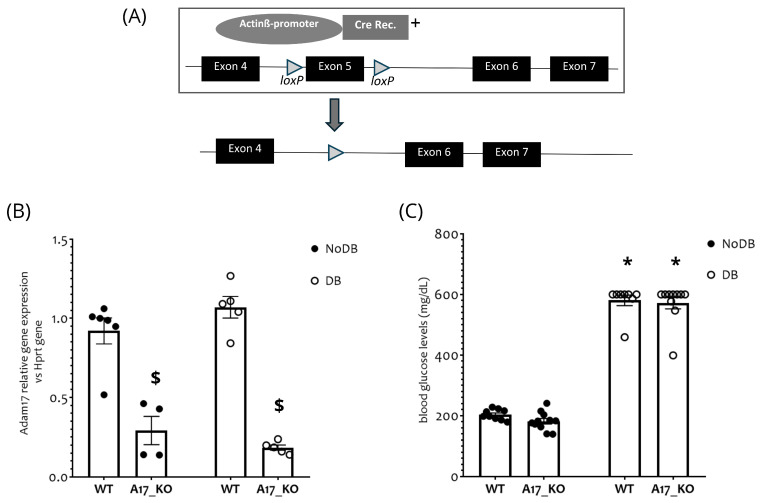
Validation of tamoxifen-induced global *Adam17* deletion. (**A**) Schematic representation of the tamoxifen-inducible global *Adam17* knockout model. (**B**) *Adam17* mRNA expression levels in renal tissue, confirming effective recombination in *Adam17* knockout (KO) mice compared with wild-type (WT) controls. (**C**) Final blood glucose levels. STZ-treated mice remained diabetic throughout the 20-week follow-up. Data are presented as mean ± SEM. Groups: non-diabetic (NoDB); diabetic (DB); wild-type (WT); *Adam17* knockout (A17_KO). * *p* ≤ 0.05 vs. NoDB; $ *p* ≤ 0.05 vs. WT.

Tamoxifen administration efficiently induced global deletion of *Adam17* in adult mice. *Adam17* gene expression was markedly reduced in renal tissue from *Adam17* knockout (KO) mice compared with wild-type (WT) controls, confirming effective recombination (Figure 1B). Baseline metabolic parameters and body weight did not differ between genotypes prior to diabetes induction, indicating that global *Adam17* deletion did not affect general metabolic status under non-diabetic conditions. At study completion, both diabetic groups, WT and KO, displayed comparable hyperglycemia, significantly elevated relative to non-diabetic control (Figure 1C).

### 2.2. Global Adam17 Deletion Preserves Renal Function Despite Persistent Albuminuria

**Figure 2:**
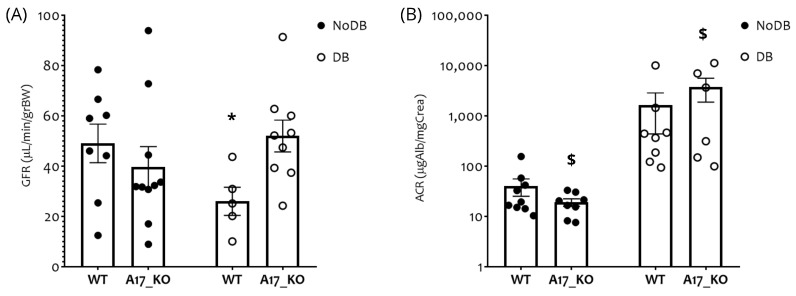
Global *Adam17* deletion preserves renal function despite persistent albuminuria. (**A**) Glomerular filtration rate (GFR) measured after 20 weeks of diabetes. (**B**) Albumin-to-creatinine ratio (ACR) in a logarithmic scale, showing increased albuminuria independently of genotype. Data are presented as mean ± SEM; 5–10 animals were included per group. Groups: non-diabetic (NoDB); diabetic (DB); wild-type (WT); *Adam17* knockout (A17_KO). * *p* ≤ 0.05 vs. NoDB; $ *p* ≤ 0.05 vs. WT.

To evaluate the impact of global *Adam17* deletion on renal function in diabetes, glomerular filtration rate (GFR) and albuminuria were assessed after 20 weeks of streptozotocin-induced diabetes. Diabetic WT mice exhibited a significant decline in GFR compared with non-diabetic controls. In contrast, diabetic *Adam17* KO mice preserved GFR, showing significantly higher filtration rates than diabetic WT animals (Figure 2A).

Albuminuria was markedly increased in both diabetic WT and *Adam17* KO mice, with no significant differences between genotypes (Figure 2B). These findings indicate a dissociation between albuminuria and renal functional decline, suggesting that global *Adam17* deletion preferentially modulates mechanisms related to loss of filtration capacity rather than glomerular permeability.

### 2.3. Adam17 Deletion Attenuates Diabetes-Induced Structural Kidney Damage

**Figure 3:**
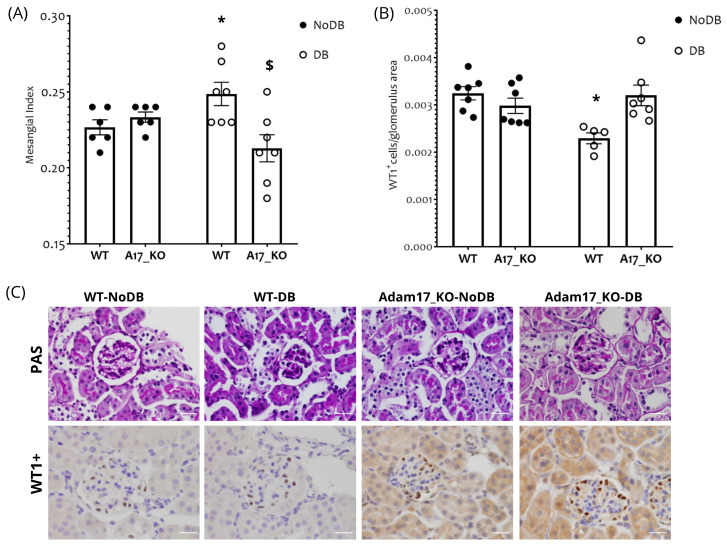
*Adam17* deletion attenuates diabetes-induced structural kidney damage. (**A**) Mesangial expansion quantification from PAS staining samples. (**B**) Podocytes detected by WT1 staining, quantified, and normalized to glomerular area. (**C**) Representative images for PAS- and WT1-stained images at 400× magnification (scale bar = 50 μm). Data are presented as mean ± SEM. Groups: non-diabetic (NoDB); diabetic (DB); wild-type (WT); *Adam17* knockout (A17_KO). * *p* ≤ 0.05 vs. NoDB; $ *p* ≤ 0.05 vs. WT.

Consistent with preserved renal function, histological analyses revealed that global *Adam17* deletion attenuated diabetes-induced structural kidney injury. Periodic acid–Schiff (PAS) staining demonstrated significant mesangial expansion in diabetic WT mice, which was markedly reduced in diabetic *Adam17* KO animals (Figure 3A,C). Furthermore, podocyte loss, assessed by WT1 immunostaining, was evident in diabetic WT kidneys but was significantly attenuated in *Adam17*-deficient mice (Figure 3B,C), indicating preservation of glomerular architecture. These results were also validated by the staining of nephrin and podocin (Figure S1).

### 2.4. Reduced Macrophage Infiltration and Chemokine-Associated Inflammation in Adam17 KO Kidneys

**Figure 4:**
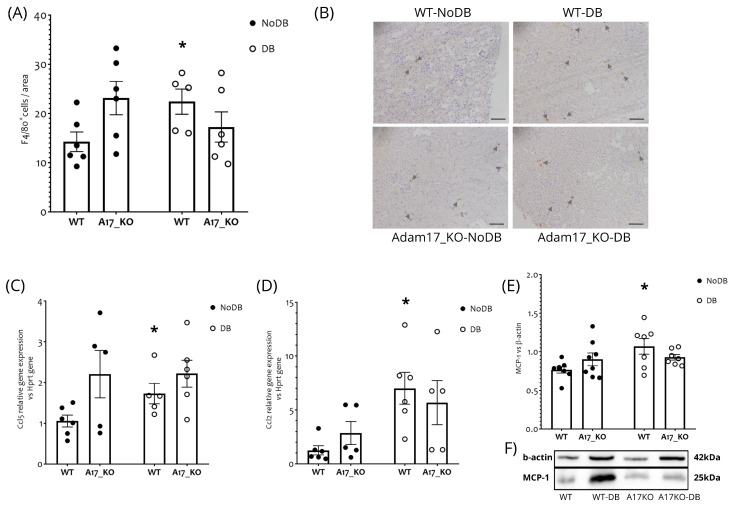
Reduced macrophage infiltration and chemokine-associated inflammation. (**A**) F4/80+ -macrophages quantified and normalized to glomerular area; (**B**) representative images of cell infiltration at 200× magnification where grey arrows indicate F4/80+ cells; (**C**) *Ccl5* expression after 20 weeks of follow up; (**D**) renal *Ccl2/MCP-1* gene expression; (**E**) MCP-1 protein levels normalized to β-actin; (**F**) representative Western blot images. Data are presented as mean ± SEM. Scale bar = 100 μm. Groups: non-diabetic (NoDB); diabetic (DB); wild-type (WT); Adam17 knockout (A17_KO). * *p* ≤ 0.05 vs. NoDB.

Renal inflammatory activation was evaluated by assessing macrophage infiltration and chemokine expression. Diabetic WT mice exhibited pronounced accumulation of F4/80-positive macrophages within the renal cortex. In contrast, diabetic *Adam17* KO mice showed similar levels as non-diabetic *Adam17* KO mice (Figure 4A,B). At the molecular level, type 1 diabetes induced an increase in *Ccl5* gene expression in WT mice (Figure 4C). Surprisingly, non-diabetic *Adam17*-KO mice displayed similarly elevated basal *Ccl5* levels compared with diabetic Adam17 KO mice, suggesting a diabetes-independent inflammatory tone associated with *Adam17* deficiency. Expression of *Mcp1* (*Ccl2*) gene was significantly increased in diabetic WT kidneys, while the differences between diabetic and non-diabetic KO mice were not statically significant. (Figure 4D). These results were better delineated at protein level where all *Adam17* KO mice showed similar expression levels despite the diabetic state (Figure 4D,E). Together, these data indicate that global *Adam17* deletion limits macrophage-associated inflammatory activation related to Mcp1.

Furthermore, given the role of Adam17 in TNF-α shedding, we wanted to assess TNF-α levels as an exploratory inflammatory marker. TNF-α concentrations in serum as the shedded form and the ones in renal tissue showed no consistent results between diabetic WT and *Adam17* KO mice (Figure S2).

### 2.5. Selective Modulation of Stress-Related Signalling Pathways in Adam17-Deficient Kidneys

**Figure 5:**
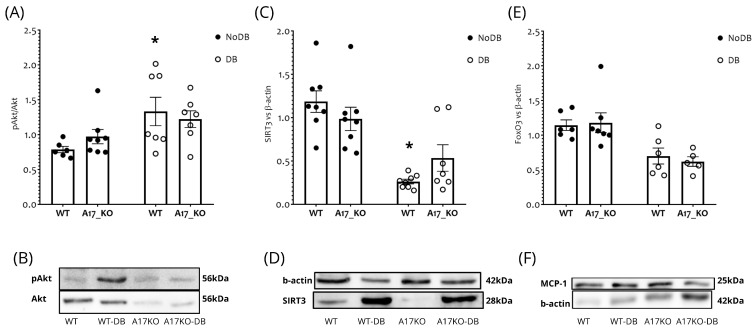
Selective modulation of stress-related signaling pathways. (**A**,**B**) p-Akt/Akt analysis and representative Western blot images for pAkt and Akt; (**C**,**D**) SIRT3 protein normalized to β-actin and representative Western blot images; (**E**,**F**) FOXO3 protein bands normalized to β-actin and representative Western blot images. Data are presented as mean ± SEM. Groups: non-diabetic (NoDB); diabetic (DB); wild-type (WT); *Adam17* knockout (A17_KO). * *p* ≤ 0.05 DB vs. NoDB.

To explore intracellular signalling mechanisms associated with *Adam17* deletion, key pathways involved in cellular stress and survival were analysed. Diabetes induced marked activation of the PI3K/Akt signalling pathway in WT kidneys, as evidenced by increased Akt phosphorylation. This response was significantly attenuated in diabetic *Adam17* KO mice where both groups had similar values (Figure 5A,B).

Analysis of mitochondrial and oxidative stress regulators revealed a more selective pattern of modulation. SIRT3 expression was partially preserved in *Adam17*-deficient kidneys compared with diabetic WT controls (Figure 5C,D), whereas FoxO3 expression and activation remained largely unchanged (Figure 5E,F).

### 2.6. Global Adam17 Deletion Limits Fibrotic Remodeling in Diabetic Kidneys

**Figure 6:**
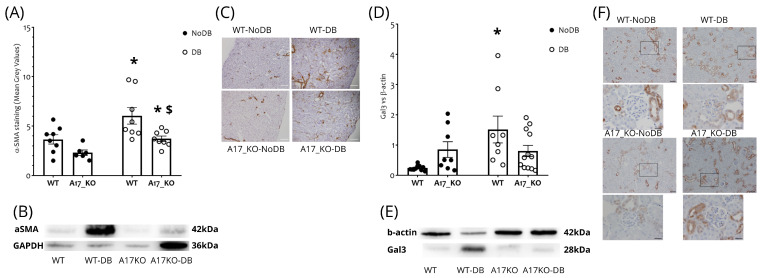
Global *Adam17* deletion limits fibrotic remodeling. (**A**,**C**) Fibrosis due to α-SMA staining and quantification on cortex kidney sections as shown in the microphotographs at 200× magnification, Scale bar = 100 μm; (**B**) α-SMA protein bands compared with GAPDH bands in a representative Western blot image; (**D**,**E**) galectin-3 protein bands normalized to β-actin and representative bands in Western blot images; (**F**) Representative microphotographs showing Gal3 immunohistochemical staining at 100× and 400× magnification. The squared area in the 100× image indicates the region shown at higher magnification (400×) to illustrate the staining pattern in greater detail. Scale bars = 50 μm and 20 μm, respectively. Data are presented as mean ± SEM. Groups: non-diabetic (NoDB); diabetic (DB); wild-type (WT); *Adam17* knockout (A17_KO). * *p* ≤ 0.05 vs. NoDB, ^$^ *p* ≤ 0.05 vs. WT-DB.

Fibrotic remodeling was markedly reduced in diabetic *Adam17* KO mice. Immunohistochemical analysis demonstrated significant upregulation of α-smooth muscle actin (α-SMA) and galectin-3 in diabetic WT kidneys, reflecting myofibroblast activation and fibrotic progression. In contrast, expression of both markers was significantly attenuated in *Adam17*-deficient mice (Figure 6).

Collectively, these results demonstrate that global *Adam17* deletion confers significant protection against diabetes-associated kidney injury by preserving renal function, attenuating structural damage, limiting macrophage-associated inflammation, selectively modulating stress-related signalling pathways, and reducing fibrotic remodelling.

## 3. Discussion

In the present study, we demonstrate that tamoxifen-inducible global *Adam17* deletion markedly attenuates diabetes-associated kidney injury in a murine model of type 1 diabetes. Global *Adam17* deficiency preserved renal function while attenuating structural injury, inflammatory recruitment, stress-related signalling, and fibrotic remodelling despite persistent hyperglycaemia and ongoing albuminuria. These findings extend previous observations obtained in cell-specific *Adam17* deletion models and support the concept that Adam17 contributes to diabetic kidney disease through integrated multicellular and systemic mechanisms beyond compartment-restricted effects [22,23,24].

Notably, preservation of glomerular filtration rate occurred in the absence of significant improvement in albuminuria, highlighting the partial dissociation between glomerular permeability markers and functional renal decline. This observation is particularly relevant given the increasing recognition of non-albuminuric phenotypes of diabetic kidney disease and supports the concept that inflammatory and fibrotic pathways may contribute to progressive renal dysfunction beyond glomerular barrier alterations alone [1,5]. In this context, our findings suggest that global *Adam17* deletion modulates coordinated pathogenic responses involved in renal injury progression rather than exclusively affecting glomerular barrier dysfunction [4,7].

Chronic inflammation plays a central role in the progression of diabetic kidney disease, contributing to both structural damage and fibrotic remodeling [2,3,4,6]. In the present study, global *Adam17* deletion was associated with reduced renal macrophage infiltration and decreased expression of chemokines involved in monocyte recruitment, including MCP-1 and CCL5. Importantly, our findings support a role for Adam17 in regulating macrophage-associated inflammatory recruitment rather than a generalized immune response. While we did not assess macrophage activation states or functional phenotypes, the reduction in macrophage accumulation is consistent with a diminished inflammatory microenvironment that may limit subsequent tissue injury and fibrogenesis [28,29,30,31].

In addition to its effects on inflammatory recruitment, *Adam17* deletion selectively modulated intracellular stress-related signaling pathways. Diabetes-induced activation of the PI3K/Akt pathway, which has been implicated in maladaptive repair responses and fibrotic progression in chronic kidney disease [7,8,32,33], was attenuated in *Adam17*-deficient kidneys. In contrast, regulators of mitochondrial and oxidative stress exhibited a more nuanced pattern of modulation, with partial preservation of SIRT3 expression and no significant changes in FoxO3 signaling. These findings suggest that Adam17 does not act as a global regulator of cellular stress responses, but rather modulates specific signaling pathways in a context-dependent manner [9,34,35].

Fibrotic remodeling emerged as one of the most robustly affected processes following global *Adam17* deletion. Reduced expression of α-smooth muscle actin and galectin-3 indicates attenuation of myofibroblast activation and extracellular matrix remodeling in diabetic *Adam17*-deficient mice. Fibrosis represents a final common pathway in chronic kidney disease progression, integrating inflammatory, metabolic, and cellular stress signals [25,36,37,38,39]. Our findings therefore support a role for Adam17 as a molecular link between chronic inflammatory activation and fibrotic progression in the diabetic kidney, providing a mechanistic basis for the observed preservation of renal function [26,27,40,41].

The pleiotropic nature of Adam17 raises important considerations regarding its role across different tissues and disease contexts. Adam17 regulates the shedding of multiple substrates involved in inflammation, growth factor signaling, and cell–cell communication, and its functional consequences are highly dependent on cellular and organ-specific settings [10,11,12,13,14,15,16,17,18]. Accordingly, divergent effects of *Adam17* deletion have been reported across experimental models, underscoring the context-dependent actions of this protease [42]. In line with this complexity, previous strategies aimed at systemic or catalytic site inhibition of Adam17 have faced important limitations, largely related to limited selectivity, tolerability issues, and interference with homeostatic shedding functions, which may contribute to heterogeneous or context-dependent outcomes across disease models [43,44,45]. In addition to inflammatory and fibrotic pathways, Adam17 is known to regulate ACE2 shedding, thereby potentially influencing RAAS balance in diabetic kidney disease [22]. Although ACE2 activity and RAAS components were not directly assessed in the present study, modulation of this axis may represent an additional mechanism contributing to the renoprotective phenotype observed after global *Adam17* deletion.

Our findings support the concept that therapeutic benefit from Adam17 modulation is likely context-dependent and may rely on identifying downstream pathways that are particularly relevant in the diabetic kidney. In this regard, the Adam17–Klotho axis emerges as an attractive mechanistic link, as increasing evidence indicates that Adam17 can promote Klotho shedding and that disruption of this pathway may influence oxidative stress-related injury and tissue vulnerability in diabetic kidney disease [46]. Finally, while direct inhibition of Adam17 by sodium–glucose cotransporter 2 inhibitors has not been demonstrated, recent studies have suggested that some of the pleiotropic renoprotective effects of SGLT2 inhibitors may involve modulation of inflammatory and stress-related pathways converging on Adam17-related signalling including Klotho-associated mechanisms [19,47]. Although this hypothesis remains speculative, it is conceptually aligned with the renoprotective profile observed in our model.

Some limitations of this study should be considered. First, the use of a global *Adam17* knockout model precludes attribution of specific effects to individual renal or immune cell populations and does not allow complete discrimination between kidney-intrinsic mechanisms and indirect effects mediated through systemic inflammatory changes. While previous cell-specific *Adam17* deletion models have provided insight into compartment-specific pathways, the present study was designed to evaluate the integrated response to global *Adam17* deficiency in experimental diabetic kidney disease. Second, inflammatory analyses were limited to macrophage infiltration and chemokine expression, without functional characterization of immune cell phenotypes. Third, this study was performed in a murine model of type 1 diabetes, and extrapolation to other forms of diabetic kidney disease or human pathology should be made with caution. Despite these limitations, the integration of functional, structural, and molecular analyses provides a comprehensive assessment of the impact of *Adam17* deletion on diabetic kidney injury. Furthermore, only male mice were included in this study. Therefore, potential sex-specific differences in the renal response to global *Adam17* deficiency could not be evaluated and warrant investigation in future studies.

## 4. Materials and Methods

- Animals and generation of global *Adam17* knockout mice

A tamoxifen-inducible global *Adam17* knockout (Adam17_KO) mouse model was generated by crossing Adam17flox/flox mice (kindly provided by Dr. Raines, Washington University, Saint Louis, MO, USA) with a murine line carrying a Cre recombinase-Estrogen Receptor fusion gene under the control of β-Actin promoter (β-Actin–Cre-ER) obtained from the Jackson Laboratory (Bar Harbor, ME, USA), allowing inducible deletion of *Adam17* in all cell types. Adam17flox/flox mice carried loxP sites flanking exon 5 of the *Adam17* gene [48], and Cre-mediated recombination resulted in excision of this exon, producing a non-functional transcript. All mice were genotyped using classical PCR technique in DNA isolated from ear clippings. Recombination was induced by tamoxifen administration in 10-week-old mice. Wild-type (WT) littermates lacking loxP sites but receiving identical tamoxifen treatment were used as controls. Successful *Adam17* deletion was confirmed in renal tissue by qPCR. All experiments were performed in male mice on a C57BL/6 background. Animals were housed under standard conditions with free access to food and water. All experimental procedures were conducted in accordance with institutional and national guidelines for animal care and use (CEA-OH/10652).

- Experimental design and induction of diabetes

Type 1 diabetes was induced in 12-week-old mice by two intraperitoneal injections of streptozotocin (Merck/Sigma-Aldrich, St. Louis, MO, USA), (150 mg/kg body weight) administered one week apart to 4 h-fasted animals [49]. Diabetes induction was confirmed by non-fasting blood glucose measurements, and mice with blood glucose levels exceeding 250 mg/dL during the first four weeks after STZ administration were considered diabetic. Diabetic and non-diabetic mice were followed for 20 weeks after diabetes induction. Each study group included 10 animals. After this period, animals were euthanized under deep anaesthesia [49], blood samples were collected by cardiac puncture, and kidneys were perfused with cold phosphate-buffered saline prior to removal. Renal tissue was processed for histological and molecular analyses.

- Assessment of renal function and metabolic parameters

Renal function was evaluated at the end of the study by measuring glomerular filtration rate (GFR) using fluorescein isothiocyanate (FITC)-inulin (Merck/Sigma-Aldrich, St. Louis, MO, USA) clearance following a single bolus injection. GFR values were expressed as μL/min/g body weight [50]. Albuminuria was assessed in morning spot urine samples collected during the final week of follow-up. Urinary albumin concentrations were measured by ELISA (Albuwell M, Exocell, Philadelphia, PA, USA) and creatinine levels were determined using a colorimetric assay (Creatinine Companion, Exocell, Philadelphia, PA, USA). The albumin-to-creatinine ratio (ACR) was calculated and expressed as μg albumin per mg creatinine [51]. Investigators were blinded to genotype during functional assessments.

- Histological and immunohistochemical analyses

Kidney tissues were fixed in formalin and embedded in paraffin for histological evaluation. Periodic acid–Schiff (PAS) staining was performed to assess glomerular morphology and mesangial matrix expansion. The mesangial index was calculated as the ratio of mesangial area to total glomerular tuft area using ImageJ v1.52 software [52]. Immunohistochemical analyses were performed to evaluate podocyte number (WT1), macrophage infiltration (F4/80), myofibroblast activation (α-smooth muscle actin, α-SMA), fibrotic remodelling (galectin-3), nephrin and podocin. Positive staining was quantified in a predefined number of fields per animal, and analyses were conducted in a blinded manner.

- Gene expression analysis

Total RNA was isolated from renal cortex tissue with Tripure Isolation Reagent (Sigma-Aldrich, St. Louis, MO, USA) and reverse-transcribed into cDNA using the High Capacity cDNA RT Kit (ThermoFisher Scientific, Waltham, MA, USA) [53]. Gene expression levels of *Adam17*, monocyte chemoattractant protein-1 (*Mcp1*/*Ccl2*), and chemokine (C–C motif) ligand 5 (*Ccl5*) were quantified by real-time PCR using LightCycler^®^480 SYBR Green I Master Mix (Roche, Basel, Switzerland). Hypoxanthine phosphoribosyltransferase 1 (*Hprt*) was used as a housekeeping gene for normalization. Relative gene expression was calculated using the 2^−ΔΔCt^ method (Table S1).

- Protein expression analysis

Protein extracts from renal cortex tissue homogenized in extraction buffer containing 50 mM HEPES, pH 7.4, 150 mM NaCl, 0.5% Triton X-100, 0.025 mM ZnCl2, (all from Merck/Sigma-Aldrich, St. Louis, MO, USA) 0.1 mM Pefabloc SC Plus (Roche, Basel, Switzerland), EDTA-free protease inhibitor cocktail tablet (Roche, Basel, Switzerland), and phosphatase inhibitor cocktail (Merck/Sigma-Aldrich, St. Louis, MO, USA) were analyzed by Western blotting. Fifteen μg of total protein was used to assess expression of phosphorylated and total Akt, MCP-1, galectin-3, SIRT3, and FoxO3. Protein expressions were quantified by pixel intensity and normalized to appropriate loading controls (Table S2).

- Tumor necrosis factor-α measurements

Tumor necrosis factor-α (TNF-α) levels were measured in serum and renal cortex homogenates using a commercially available ELISA kit according to the manufacturer’s instructions Kit (R&D Systems, Minneapolis, MN, USA). TNF-α was assessed as an exploratory inflammatory marker.

- Statistical analysis

Data are presented as mean ± standard error (SE). Statistical analyses were performed using one-way ANOVA for normally distributed data or non-parametric Kruskal–Wallis and Mann–Whitney tests when appropriate. A *p*-value < 0.05 was considered statistically significant.

## 5. Conclusions

In conclusion, our results demonstrate that global deletion of *Adam17* protects against diabetes-associated renal injury by attenuating inflammation, selectively modulating stress-related signaling pathways, and limiting fibrotic remodeling, ultimately preserving renal function. These findings extend previous cell-specific studies and highlight the context-dependent role of Adam17 in diabetic kidney disease, supporting the need for refined and tissue-aware therapeutic strategies targeting this protease.

## Data Availability

The original contributions presented in this study are included in the article/Supplementary Materials. Further inquiries can be directed to the corresponding author.

## Supplementary Materials

The following supporting information can be downloaded at https://www.mdpi.com/article/10.3390/ijms27146136/s1.

## Abbreviations

The following abbreviations are used in this manuscript:

ACR	Albumin-to-creatinine ratio
Adam17	A disintegrin and metalloproteinase 17
CCL2/MCP1	Chemokine (C-C motif) ligand 2//Monocyte chemoattractant protein 1
CCL 5	Chemokine (C-C motif) ligand 5
DKD	Diabetic kidney disease
EGFR	Epidermal growth factor receptor
FITC	Fluorescein isothiocyanate
Gal3	Galectin-3
GAPDH	Glyceraldehyde 3-phosphate dehydrogenase
GFR	Glomerular filtration rate
HPRT	Hypoxanthine phosphoribosyltransferase 1
KO	Knockout
PAS	Periodic acid–Schiff
PI3K/Akt	Phosphoinositide 3-kinase/Protein kinase B
SIRT3	Sirtuin 3
α-SMA	α-smooth muscle actin
STZ	Streptozotocin
TACE	Directory of open access journals
TNF-α	Tumor necrosis factor α
WT	Wild-type
WT1	Wilm’s tumor 1
