# Supplementary material for: Global Adam17 Deficiency Preserves Renal Function and Modulates Integrated Pathogenic Responses in Experimental Diabetic Kidney Disease
Source: Int J Mol Sci. 2026 Jul 9;27(14):6136. doi: 10.3390/ijms27146136 (PMC13410776; doi:10.3390/ijms27146136)
Supplement: Supplementary file 1 [file ijms-27-06136-s001.zip › ijms-4340936-supplementary.pdf]

Supplementary information

Figure S1. Nephrin and podocin localization in glomeruli. Both podocyte markers were detected in NoDB groups. In WT-DB animals, staining appered discontinuous and reduced, this was not observed in the Adam17_KO diabetic group. A) Representative images showing nephrin localization in glomeruli from the four study groups. B) Representative images showing podocin localization in glomerili from the four study groups. Original magnification, 400X. Groups: non-diabetic (NoDB); diabetic (DB); wild-type (WT); Adam17 knockout (Adam17_KO).

A)

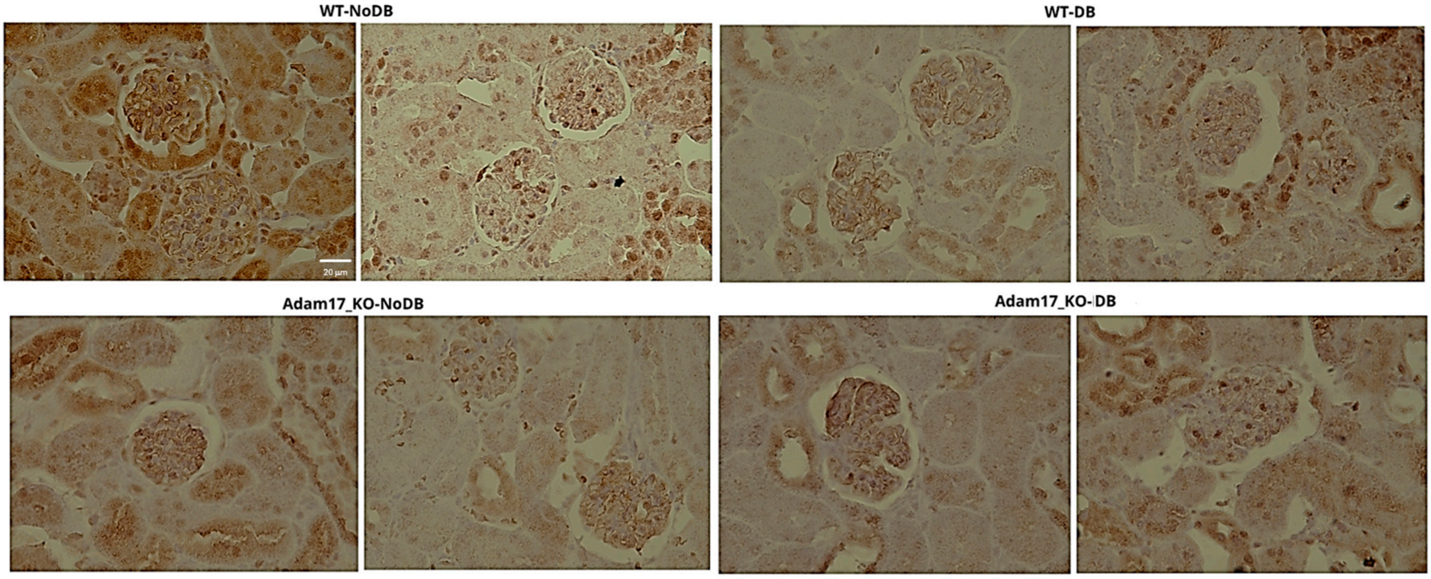

B)

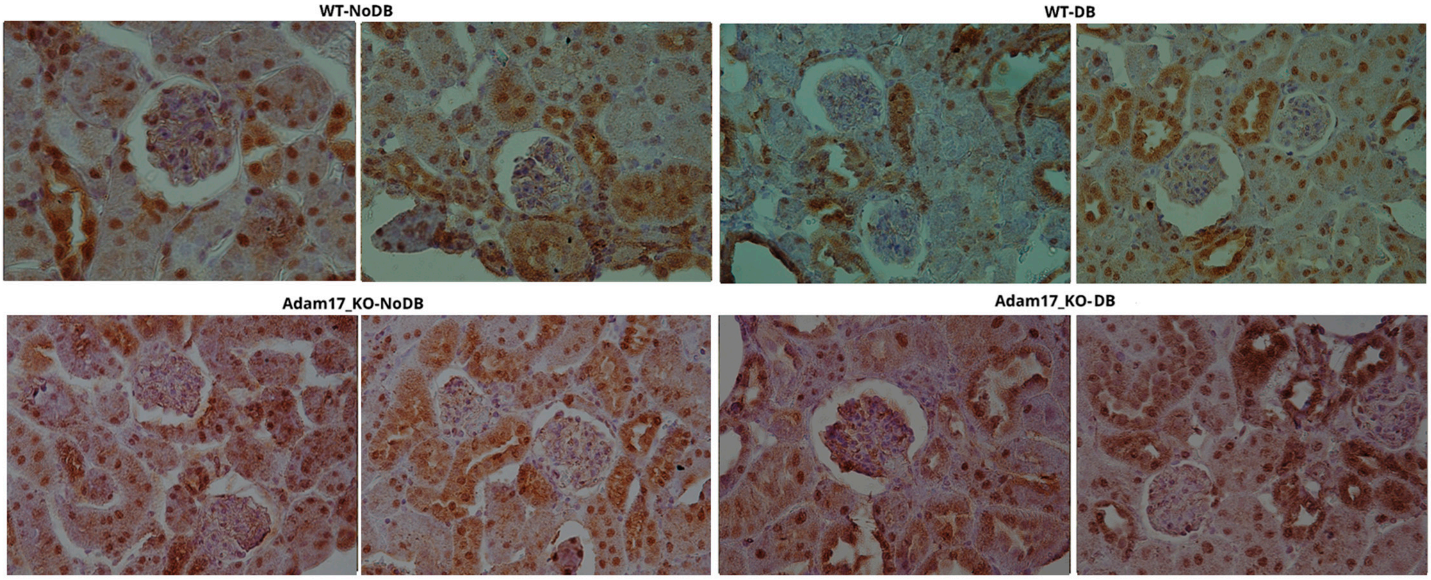

Figure S2. Influence of diabetes and Adam17 deletion on circulating and kidney cortex TNF-α. A) Serum TNF-α quantified by ELISA; B) TNF-α levels in kidney cortex homogenates. Data are presented as mean ± SEM. Groups: non-diabetic (NoDB); diabetic (DB); wild-type (WT); Adam17 knockout (A17_KO).

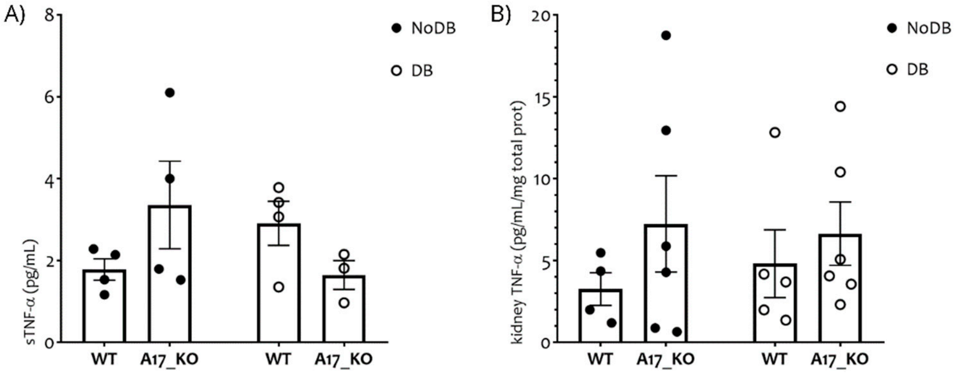

Table S1. List of primers used for the analysis of the genes. The table is divided according the type of primers, located at genomic or at cDNA sequences used for both processes.

Target gene	Primer Forward	Primer Reverse
genomic_actinCre	5′ CCTGGCGATCCCTGAACATGTCC 3′	5′ CTCTAGAGCCTCTGCTAACC 3′
genomic_Adam17Fl_F	5′ ATAGGGAGCCAAGTGTGATGG 3′	5′ CACATACTTGCCTACAAGCCAG 3′
Adam17	5′ GGCAGAATATAACGTAGAGCCACT 3′	5′ CTTCAGACTTATACACCAGC 3′
Mcp1/Ccl2	5′ AGGTCCCTGTCATGCTTCTG 3′	5′ CGTTAACTGCATCTGGCTGA 3′
Ccl5	5′ CTGCTGCTTTGCCTACCTCT 3′	5′ GTGACAAACACGACTGCAAGAT 3′
Hprt	5′ TGTTGTTGGATATGCCCTTG 3′	5′ AATGACACAAACGTGATTCAAA 3′
Adam17	5′ GGCAGAATATAACGTAGAGCCACT 3′	5′ CTTCAGACTTATACACCAGC 3′

Table S2. Antibodies used for immunohistochemistry and for Western blot (WB). Secondary antibodies for the WB were Peroxidase AffiniPure Donkey Anti-Rabbit IgG (H+L) and Peroxidase AffiniPure Donkey Anti-Mouse IgG (H+L) (Jackson Immunoresearch). BSA: Bovine Serum Albumin; GS: Goat Serum; NFM: Non-Fat Milk diluted in TBS-T 0.1%.

	Source	Cat. Number	Company	Working dilution
**Immunohistochemistry**				
F4/80	rat	123101	Biolegend	1:500 in 3%BSA/3%GS
WT1	rabbit	12609-1-AP	Proteintech	1:1000 in 3%BSA/3%GS
α-SMA	mouse	A-2547	Sigma-Aldrich	1:1000 in 3%BSA/3%GS
nephrin	rabbit	CAB3048	AssayGenie	1:200 in 1%BSA/3%GS
podocin	rabbit	CAB17337	AssayGenie	1:200 in 1%BSA/3%GS
**Western Blot**				
Galectin3	mouse	126701	Biolegend	1:500 in 2.5% BSA
pAKT (Ser473)	rabbit	9271 S	Cell Signaling Technology	1:1000 in 2.5% BSA
Akt	rabbit	9272 S	Cell Signaling Technology	1:2000 in 2.5% BSA
MCP1/CCL2	mouse	TA336914	Origene	1:2000 in 2.5% NFM
SIRT3	rabbit	CAB7307	Assay Genie	1:3000 in 2.5% NFM
FoxO3	rabbit	CAB0102	Assay Genie	1:3000 in 2.5% NFM
α-SMA	mouse	A-2547	Sigma-Aldrich	1:1000 in 2.5% NFM
Galectin3	mouse	126701	Biolegend	1:1000 in 2.5% NFM
ß-actin	mouse	A1978	Sigma-Aldrich	1:20000 in 2.5% NFM
Gapdh	mouse	sc-32233	Santa Cruz Biotechnology	1:20000 in 2.5% NFM
